# Remarks on Mr. Knowles's Cases

**Published:** 1811-11

**Authors:** 


### *To the Editors of the Medical and Physical Journal.*

Gentlemen,

In looking over your Journal for last month, I noticed a paper from the pen of Mr. Knowles, on the subject of diarrhœa; now, as I am a sincere lover of the profession, I am always glad to see any new road pointed out which may lead to a more successful mode of treating diseases, and the improvement of the medical art; but if medical men will relate cases which contain neither novelty, interest, or amusement, they certainly come under the lash of public opinion; and I would ask Mr. Knowles what there is in this case of either? Is there any thing in the treatment of it different from the common routine of practice; or are obstinate diarrhœas of "uncommon occurrence," as he has in the outset of his paper declared them to be? They are, I am sure, met with by every practitioner. Mr. Knowles supposes that "some morbific matter, producing irritation in the bowels, was the predisposing cause of this disease;" but this is impossible. It might be the *exciting* cause, but the predisposition must be sought for in something else: I should be inclined to think in nervous irritation, and a deranged state of the digestive organs, connected with a faulty action of the liver and irregular secretion of bile; but Mr. Knowles's account of the case is extremely defective: he has not even noticed the *colour* of the stools, a circumstance of the greatest importance, as Mr. Abernethy has very justly remarked, in all disorders of the chylopoietic viscera. Had he given small alterative doses of mercury, in all probability he would not have experienced the "long and tedious trial" which he did. I shall forbear making any further remarks on this case, and beg leave to offer a few observations on some other of Mr. Knowles's papers, in different Numbers of your Journal.

In April, 1810, "On the management of Leeches," he has sagaciously told you that he "believes there is no *insensible* membrane on the mouth of a leech;" that in order to make a leech disgorge herself of blood, you should gently draw it through your fingers!!; and, to keep them alive, that *you should put them into a bottle of water, covered over at the mouth with a piece of leather, having holes pricked in it to admit air!!*

In your next Number, I again find him on a most curious subject, "Plica Polonica," in which he states "being in the shop of Mr. Rogers, printer, of this town, (Newmarket), he presented to me, for inspection, a plica nine feet long, which

 is is now in his possession." I have also seen this "plica," as he is pleased to term it; and there is not the least reason whatever to suppose that it had any thing to do with the disease in question, being simply a morbid growth of the hair, unattended by any of the symptoms which precede or characterize the genuine disease; as general lassitude and heaviness, pains in different parts of the body, particularly of the head and eyes, and some degree of febrile affection; for he has said nothing about them: neither were the hairs enlarged in their diameters, or glued together with the viscid matter which issues from the roots in the true trichoma; as may be gathered from the following passage of his paper: "She was in the habit of running a comb through it, *to keep it together*; and she used to consider it as a great ornament." Finally, none of the dreadful consequences would have followed, had this lock of hair been extirpated at any time; or had it been the genuine plica polonica: for, in opposition to the authority of M. de la Fontaine, we have the following fact, which occurred at Breslau, and communicated by Dr. de Carro, of Vienna, in a letter to the editors of the Bibliothéque Britannique.

"Some years ago, one third of the recruits of the regiments of artillery brought from South Prussia were attacked with plica polonica. An order was received from Berlin to send to that city all those that were infected, and to take care that the disease was not communicated to others. This order, it appears, was not agreeable to the commanders of companies, as it would have occasioned the loss of at least 200 young soldiers. M. Hand, surgeon-major to the artillery regiments, became mediator in the case; he made the recruits be brought on the ramparts, and ordered that a general shaving should be made. In a little time a pile of plica was accumulated; these trophies were then cast into a ditch, and the heads of the men carefully washed with soap and water daily, for some weeks. By this simple method of treatment, these dirty Polanders were speedily transformed into good soldiers, without having in the least suffered by the loss of this precious ornament of their head."

In the Number for August, 1810, I find a "Case of Ascites" successfully treated by Mr. Knowles. Now, gentlemen, as I know you are sometimes liable to be imposed upon by the relation of cases with a successful mode of treatment subjoined, which have nevertheless terminated in a very different manner; and as I have great reason to suppose that you have been so in the present instance, I am exceedingly anxious to detect it, if possible. I am much inclined to think that the subject of this case was a woman living at Thetford, and under the care of two eminent practitioners of that place, who, as Mr. Knowles

Knowles has remarked, did differ in opinion with respect to the tumor of the hypochondrium. She had also a brother, a medical gentleman, who communicated, in a letter to Mr. Knowles, a statement and history of her case; but so far from this lady ever being under his care at any time, I believe he never saw her but once; and I can also most confidently affirm, that he never met the two medical gentlemen who attended her: nor did she ever at any one time take a single grain of medicine of his prescribing. The powers of the constitution being gradually worn down, she died, poor woman, contrary to Mr. Knowles's statement, who has said that she recovered, and was afterwards the mother of two children. The treatment of ascites has always been of acknowledged difficulty; and I would ask, whether it appears probable that Mr. Knowles should have succeeded so easily by a few doses of calomel and digitalis, after the united efforts of two medical men, of great experience, had been exerted without benefit to the patient, who had more than once undergone the operation of paracentesis.

Mr. Harrold, whose name I am at liberty to mention on this occasion, communicated the case of his sister to Mr. Knowles, in 1809. Mr. Knowles states his case to have happened in 1803; but *where* did it happen? Not in the neighbourhood of Newmarket, because he was not in practice there, till the latter end of 1809, or beginning of 1810.

Finally, I may close my remarks by observing, that it is extremely improbable that Mr. Knowles should meet with a case so exactly agreeing, both in *circumstances and date*, as to require the *very words* of Mr. Harrold's letter to designate it; for I have seen a copy of the letter to Mr. Knowles, and can affirm, that the whole of the description and history of his case, is given, word for word, from this identical letter in question.

A NORFOLK PRACTITIONER.

